# Retrospective Clinical Study on Early Prediction of Anastomotic Leak After Esophageal Cancer Resection Based on the Combination of Platelet Count and Neutrophil-to-Lymphocyte Ratio

**DOI:** 10.7759/cureus.81589

**Published:** 2025-04-01

**Authors:** Shu Wu, Linxiang Zhang, Yamen Muad, Zhong Xu, Lin Ye

**Affiliations:** 1 Department of Cardiothoracic Surgery, The First Affiliated Hospital of Chongqing Medical University, Chongqing, CHN

**Keywords:** anastomotic leak after esophageal cancer resection, combination of platelet count and neutrophil-to-lymphocyte ratio, esophagectomy, postoperative complications, systemic inflammatory response

## Abstract

Objective: The systemic inflammatory response may influence the occurrence of postoperative complications. This study aimed to evaluate the predictive potential of combining platelet count and neutrophil-to-lymphocyte ratio (COP-NLR) for esophagogastric anastomotic leak (AL) following esophageal cancer surgery.

Methods: We enrolled patients who developed AL after radical surgery for esophageal cancer and those who did not develop AL after the surgery at the First Affiliated Hospital of Chongqing Medical University, China, from June 2019 to February 2022. We analyzed the correlation between AL and several risk factors, including COP-NLR. Patients were categorized as COP-NLR 2 if both platelet count and neutrophil-to-lymphocyte ratio (NLR) were elevated, COP-NLR 1 if either parameter was elevated, and COP-NLR 0 if neither parameter showed elevation.

Results: A total of 190 patients were included in this study. The incidence of AL after esophageal cancer surgery was 14.7%. The critical values of preoperative NLR and preoperative platelet count were 2.41 (sensitivity 48.8%, specificity 92.9%, and area under the curve (AUC) 0.728) and 186 × 10^9^/L (sensitivity 45.3%, specificity 78.9%, and AUC 0.667), respectively. According to multivariate analysis, COP-NLR was identified as an independent risk factor for AL (COP-NLR 1 vs. COP-NLR 0: odds ratio (OR) 4.98, 95% confidence interval (CI) 1.05-23.61; COP-NLR 2 vs. COP-NLR 0: OR 11.12, 95% CI 2.31-53.41).

Conclusion: COP-NLR is a new predictor for AL after esophageal cancer resection.

## Introduction

According to the global tumor profile data released by the International Agency for Research on Cancer in 2020, esophageal cancer ranks seventh in incidence among all malignant tumors, representing a widespread health threat. More prominently, it ranks sixth among all malignant tumors in terms of mortality, underscoring the significant challenge this disease poses to global public health [1]. Surgery remains the preferred treatment option and occupies a central position in the clinical treatment of esophageal cancer. However, the surgical procedure is complex and delicate. It involves removing several key organs, including the esophagus, a portion of the proximal stomach, and the mediastinal and abdominal lymph nodes. This surgery usually adopts esophagogastric anastomosis to reconstruct the upper gastrointestinal tract, aiming to restore the patient's physiological function. Complications after esophageal cancer surgery are common. Approximately 65% of patients undergoing esophageal cancer resection are challenged by various types of complications, considerably threatening their recovery process and quality of life [2]. Esophageal cancer patients often face a series of complications after surgery, including anastomotic leak (AL), pulmonary infection, recurrent laryngeal nerve dysfunction, and chylothorax. These complications exacerbate patient suffering and adversely affect postoperative recovery.

It has been reported that the incidence of AL after esophageal cancer surgery is approximately 11.4% [3]. According to a report by the Esophagectomy Complications Consensus Group (ECCG), the incidence of AL after esophageal cancer resection is on the rise, increasing from 11.7% to 13.1%[4]. This change highlights that postoperative AL is one of the common complications of esophageal cancer surgery. The statistical results of the China Esophageal Cancer Survey Database in 2020 show that the incidence of AL after esophageal cancer surgery in China is around 4.6% [5]. As one of the most dangerous complications following esophageal cancer surgery, AL not only prolongs hospitalization and increases financial burdens but also adversely affects surgical outcomes and long-term prognosis [6]. AL can rapidly deteriorate, inducing thoracic infections that can escalate into serious conditions such as empyema and mediastinal infection. These complications, if not promptly and effectively controlled, may ultimately lead to life-threatening conditions such as septic shock and multi-organ failure, thus significantly increasing the risk of death after esophageal cancer surgery [7]. Kassis et al. analyzed the 30-day mortality rate of 7,595 patients who underwent esophageal cancer resection and found that the mortality rate remained as low as 3.1% in patients who did not develop AL, whereas it increased significantly to 7.2% in patients who did [8]. These data strongly suggest that the occurrence of AL can remarkably increase perioperative mortality [9]. It has also been reported that with regard to the long-term prognosis of esophageal cancer, severe AL significantly reduces overall survival and disease-free survival [10].

In clinical practice, surgeons have observed that complications arising after esophageal cancer resection are often inextricably linked to the inflammatory response. As such, the role of the host inflammatory response is an increasingly important focus of research. A series of biomarkers based on the systemic inflammatory response can effectively predict prognosis. These include neutrophil-to-lymphocyte ratio (NLR), platelet-to-lymphocyte ratio (PLR), platelet count, and combination of platelet count and neutrophil-to-lymphocyte ratio (COP-NLR). In recent years, multiple studies have revealed a significant correlation between COP-NLR and the clinical outcomes of various cancers [11-23]. To the best of our knowledge, however, no studies have elaborated on the potential association between COP-NLR and complications after esophageal cancer resection.

The primary objective of this study was to evaluate the predictive potential of COP-NLR for AL after esophageal cancer surgery.

## Materials and methods

Research subjects

We selected 190 patients who underwent radical surgery for esophageal cancer at the First Affiliated Hospital of Chongqing Medical University, China, from June 2019 to February 2022, including those who developed AL and those who did not during the same period. We collected the demographic information and clinical data of these patients, including, age, gender, height, weight, body mass index (BMI), smoking habits (specifically the number of cigarettes smoked each day), alcohol consumption, history of diabetes mellitus, and other basic information. Moreover, we recorded specific details related to the surgery, such as the anastomosis method and surgical site, as well as the number of key serological indicators, including hemoglobin concentration, platelet count, neutrophil count, lymphocyte count, and albumin level. To ensure data completeness and accuracy, any cases with missing information were excluded from this study.

The Ethics Committee of the First Affiliated Hospital of Chongqing Medical University approved this study (approval number: K2023-655).

Definition of anastomotic leak (AL)

According to the ECCG, AL is defined as “a full-thickness defect of the esophagus, anastomotic site, staple line, or duct, regardless of its manifestation or differentiation method.” Based on the different treatment strategies, the ECCG classifies AL into three types: type I, which is less severe and does not require adjusting the current clinical treatment plan; type II, which requires intervention but is not yet severe enough to require surgical intervention; and type III, which is the most severe type and requires additional surgical intervention for proper management [11].

Diagnostic criteria for anastomotic leak (AL)

Clinical Manifestations of Cervical Anastomotic Leak (AL)

Patients show characteristics of inflammatory response in the skin of the neck, such as the presence of red and swollen areas, obvious tenderness, and subcutaneous emphysema. In addition, foul-smelling pus is often discharged from the fistula. After removing the surgical sutures, it can be observed that the pus is mixed with food residue. Some patients may also have systemic symptoms such as fever.

Clinical Manifestations of Thoracic Anastomotic Leakage

Patients have persistent high fever, cough up a large amount of thick phlegm, and experience severe chest pain, difficulty breathing, and hydropneumothorax on the surgical side. Some patients experience toxic shock.

Diagnosis can be made if any of the following and/or clinical manifestations of AL are present [12]: (1) a clear fistula can be observed after removing the sutures from the cervical incision; (2) chest X-ray or chest CT examination reveals wrapped effusion or hydropneumothorax on the surgical side; (3) contrast agent spillage can be found during the esophagography examination; (4) endoscopy directly shows the existence of fistula; (5) blue liquid flows out of the drainage tube after administering methylene blue solution; (6) drainage of food residue or foul-smelling liquid through chest drainage tubes can serve as indirect diagnostic evidence.

Inclusion and exclusion criteria

Inclusion Criteria

Patients (1) who met the diagnostic criteria for esophageal cancer according to the 2024 Edition of the Chinese Society of Clinical Oncology (CSCO） Esophageal Cancer Diagnosis and Treatment Guidelines and were diagnosed with esophageal malignancy confirmed by pathological tissue biopsy; (2) who underwent radical surgery for esophageal cancer at the First Affiliated Hospital of Chongqing Medical University; and (3) who had records of routine blood tests (neutrophils, lymphocytes, and platelets) on the day of admission were included.

Exclusion Criteria

Patients (1) receiving neoadjuvant chemotherapy or radiotherapy; (2) with other malignant tumors or distant metastasis before surgery; (3) undergoing emergency surgery; (4) with severe cardiopulmonary and liver and kidney dysfunction before surgery; and (5) with incomplete clinical electronic medical records or paper medical records were excluded.

Definition of predictor variables and calculation of minimum sample size

The NLR was derived by dividing the absolute neutrophil count (number/L) by the absolute lymphocyte count (number/L). The COP-NLR was calculated as follows. Patients were categorized as COP-NLR 2 if both platelet count and NLR were elevated, COP-NLR 1 if either parameter was elevated, and COP-NLR 0 if neither parameter showed elevation. There is a lack of research on the relationship between COP-NLR and postoperative complications of esophageal cancer, and COP-NLR is mostly used in cancer prognosis research. In this study, the Youden index was used to determine the cutoff values of the platelet count and NLR through receiver operating characteristic (ROC) curves. By performing the power test in R, version 4.3.1 (R Foundation for Statistical Computing, Vienna, Austria, https://www.R-project.org/), the minimum sample size required for this experiment was calculated to be 150. To ensure the stability of the study and the reliability of the results, this calculation fully took into account the dropout rate, and an expected dropout rate of 20% was included in this value.

Statistical methods

Continuous variables were expressed as medians (interquartile range), and categorical variables were expressed as counts (percentages). Univariate analysis was performed using Fisher's exact test (the chi-squared test) for the incidence of AL. To identify potential predictors of AL, a multiple logistic regression model was employed to analyze the outcome variables. The selection of predictors for the multivariate model was determined by the findings from the univariate analysis and clinical settings. The ROC curve was used to analyze statistically significant indicators, identify the cutoff values of the predictors based on the Youden index, determine the sensitivity and specificity, and calculate the area under the ROC curve (AUC) of each predictor. Moreover, the Delong test was performed to analyze the AUC of the relevant predictors. We considered p < 0.05 statistically significant in terms of determining correlations between inflammation-related indicators and AL after esophageal cancer surgery. All statistical analyses were conducted using R, version 4.3.1.

## Results

**Figure 1:**
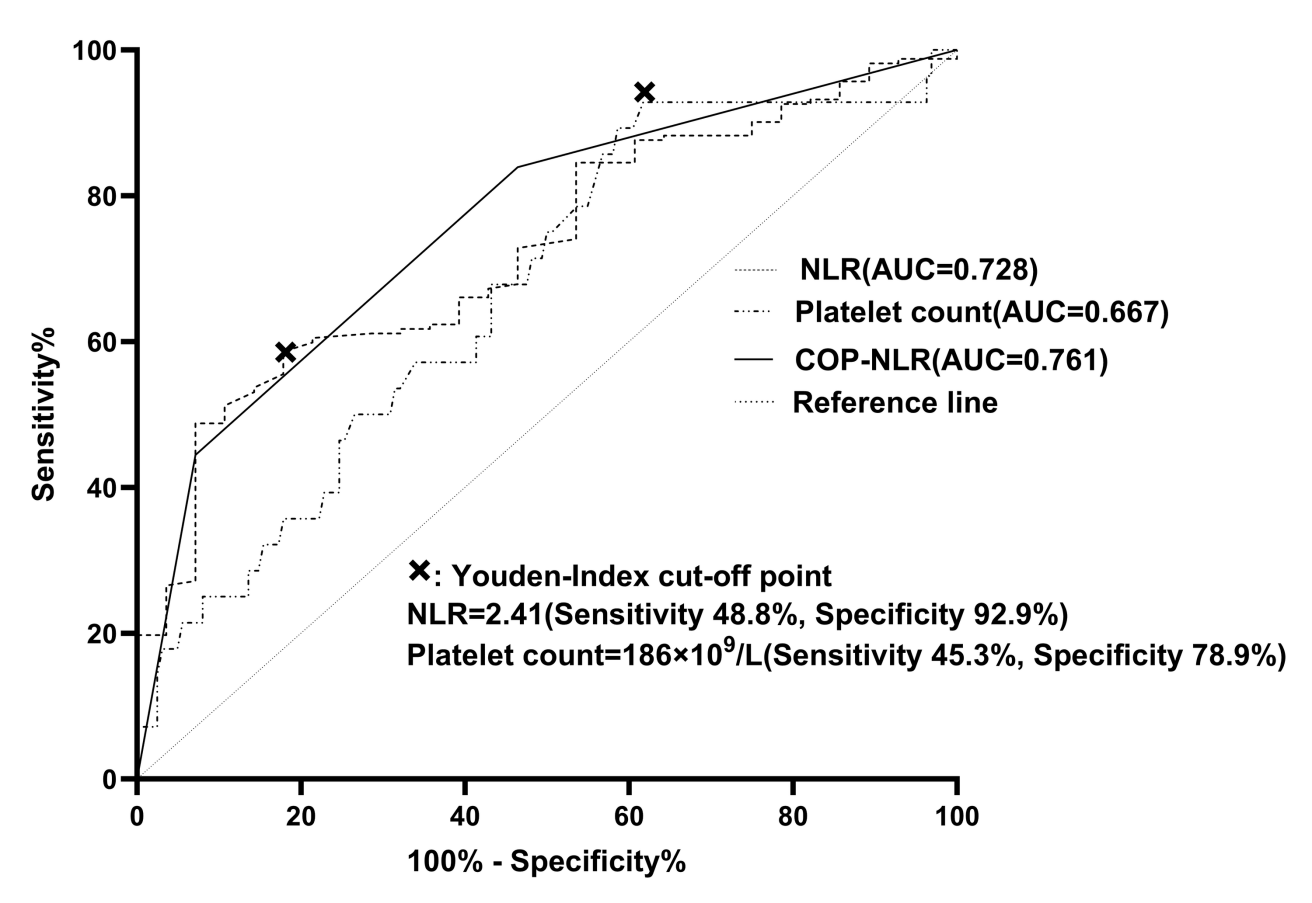
ROC curves AUC: area under the curve, ROC: receiver operating characteristic, COP-NLR: combination of platelet count and neutrophil-to-lymphocyte ratio, NLR: neutrophil-to-lymphocyte ratio.

The incidence of AL was 14.7% (n = 28/190). As shown in Figure 1, the Youden index calculation revealed that the critical value of preoperative NLR was 2.41 (sensitivity 48.8%, specificity 92.9%, and AUC 0.728), while the critical value of preoperative platelet count was 186 × 10^9^/L (sensitivity 45.3%, specificity 78.9%, and AUC 0.667). As shown in Table 2, 55 cases were categorized as COP-NLR 2, and 75 cases were classified as COP-NLR 1. Among the 75 cases classified as COP-NLR 1, 28 were determined by the NLR and 47 by the platelet counts. Univariate analysis identified significant associations between AL and key inflammatory markers. Patients with NLR ≥ 2.41 × 10⁹/L had a 3.2-fold higher AL incidence compared to those with lower counts (24.1% vs. 7.5%, p = 0.001). Patients with platelet counts ≥ 186 exhibited a 2.6-fold higher AL incidence compared to those with lower counts (20.6% vs. 8.0%, p = 0.014). Notably, the incidence of AL escalated progressively with higher COP-NLR scores, ranging from 3.3% (COP-NLR 0) to 27.3% (COP-NLR 2) (p < 0.001). Univariate analysis identified the NLR, platelet count, and COP-NLR as risk factors for AL.

**Table 1:** Patient characteristics BMI: body mass index.

Variable	n (%)
Sex	
Male	158 (83.16)
Female	32 (16.84)
Age (years)	
Range	40-83
Median	63
Current smoker	
No	55 (28.95)
Yes	135 (71.05)
Diabetes mellitus	
No	174 (91.58)
Yes	16 (8.42)
Current drinker	
No	74 (38.95)
Yes	116 (61.05)
Cervical anastomosis	
No	119 (62.63)
Yes	71 (37.37)
Anemia	
No	168 (88.42)
Yes	22 (11.58)
Hypoproteinemia	
No	182 (95.79)
Yes	8 (4.21)
BMI	
<18.5	28 (14.74)
Normal	136 (71.58)
≥25	26 (13.68)

**Table 2:** Univariate analysis of AL related to differences in demographics of all patients In this table, we list the number and proportion of AL occurrence for each factor before surgery. p < 0.05 was considered statistically significant. BMI: body mass index, COP-NLR: combination of platelet count and neutrophil-to-lymphocyte ratio, NLR: neutrophil-to-lymphocyte ratio, AL: anastomotic leak.

Factor	Non-AL	AL	n (%)	p-value
Total	162	28	14.7	
Sex				0.668
Male	136	22	13.9	
Female	26	6	18.8	
BMI				0.721
<18.5	24	4	14.3	
Normal	117	19	14	
≥25	21	5	19.2	
Current smoker				0.686
No	46	9	16.4	
Yes	116	19	14.1	
Diabetes mellitus				0.114
No	151	23	13.2	
Yes	11	5	31.3	
Current smoker				0.424
No	65	9	12.2	
Yes	97	19	16.4	
Cervical anastomosis				0.135
No	105	14	11.8	
Yes	57	14	19.7	
Anemia				0.869
No	144	24	14.3	
Yes	18	4	18.2	
Hypoproteinemia				0.178
No	157	25	13.7	
Yes	5	3	37.5	
Platelet				0.014
<186 x 10^9^/L	81	7	8	
≥186 x 10^9^/L	81	21	20.6	
NLR				0.001
<2.41	99	8	7.5	
≥2.41	63	20	24.1	
COP-NLR				0.001
0	58	2	3.3	
1	64	11	14.7	
2	40	15	27.3	

As shown in Table 3, compared to the reference group (COP-NLR 0), the risk of AL increased significantly with higher COP-NLR scores: a 4.98-fold increase for COP-NLR 1 (95% CI 1.05-23.61; p = 0.043) and an 11.12-fold increase for COP-NLR 2 (95% CI 2.31-53.41; p = 0.003). In contrast, neither diabetes mellitus (OR = 2.03, 95% CI 0.61-6.77; p = 0.250) nor anemia (OR = 2.04, 95% CI 0.57-7.30; p = 0.273) showed a statistically significant association with AL. The results suggest that inflammatory markers may be more effective than traditional metabolic factors in predicting AL. According to the multivariate analysis, COP-NLR was identified as an independent risk factor for AL.

**Table 3:** Multivariate logistic regression analysis of AL of all patients p < 0.05 was considered statistically significant. COP-NLR: combination of platelet count and neutrophil-to-lymphocyte ratio, AL: anastomotic leak, CI: confidence interval, OR: odds ratio.

Factor	β	p-value	OR (95% CI)
Diabetes mellitus			
No			1.00 (reference)
Yes	0.71	0.250	2.03 (0.61-6.77)
Anemia			
No			1.00 (reference)
Yes	0.71	0.273	2.04 (0.57-7.30)
COP-NLR			
0			1.00 (reference)
1	1.61	0.043	4.98 (1.05-23.61)
2	2.41	0.003	11.12 (2.31-53.41)

The clinical significance of COP-NLR was further assessed by comparing the ROC curves of NLR, platelet count, and COP-NLR. As shown in Table 4, ROC analysis demonstrated that COP-NLR had the highest predictive accuracy for AL, with an AUC of 0.761 (95% CI 0.677-0.845), followed by NLR (AUC = 0.728, 95% CI 0.637-0.818) and platelet count (AUC = 0.667, 95% CI 0.560-0.775). The AUC difference between COP-NLR and platelet count was statistically significant (AUC difference = 0.094, 95% CI 0.005-0.183; p = 0.043), indicating the superior discriminative ability of COP-NLR. However, no significant differences were observed between NLR and platelet count (AUC difference = 0.060, p = 0.398) or between NLR and COP-NLR (AUC difference = 0.034, p = 0.445). The nonsignificant AUC difference between NLR and COP-NLR (AUC difference = 0.034, p = 0.445) may be attributed to their shared inflammatory pathways.

**Table 4:** AUC comparison about predictive factors p < 0.05 was considered statistically significant. AUC: area under the curve, CI: confidence interval, NLR: neutrophil-to-lymphocyte ratio, COP-NLR: combination of platelet count and neutrophil-to-lymphocyte ratio.

	AUC value (95% CI)	AUC difference (95% CI)	p-value
Platelet count	0.667 (0.560-0.775)	0.060 (-0.079 to 0.199）	0.398
NLR	0.728 (0.637-0.818)		
NLR	0.728 (0.637-0.818)	0.034 (-0.119 to 0.052）	0.445
COP-NLR	0.761 (0.677-0.845)		
Platelet count	0.667 (0.560-0.775)	0.094 (0.005 to 0.183）	0.043
COP-NLR	0.761 (0.677-0.845)		

## Discussion

In this study, elevated preoperative COP-NLR scores were significantly associated with the occurrence of AL in patients after esophageal cancer resection. We also found that an elevated NLR or elevated platelet count alone was significantly associated with the occurrence of AL. To further evaluate the predictive performance of these indicators, we analyzed their ROC curves in predicting AL. Our analysis of the AUCs of the COP-NLR, NLR, and platelet count showed that COP-NLR is a more reliable predictor for the occurrence of AL.

The COP-NLR scoring system is a method for clinical prediction that utilizes systemic inflammatory responses as its basis. It combines scores assigned to the NLR and platelet count. The role of the COP-NLR score in predicting the clinical prognosis of patients with various cancers has been explored in several recent studies [13-23]. COP-NLR has demonstrated its significant value as an independent prognostic factor in many patient groups with different treatment backgrounds, providing a promising new method for predicting the prognosis of cancer patients. It has been commonly used for prognostic prediction of cancer in previous studies, where the critical values of the NLR and platelet count were usually set to 3 and 300 × 10^9^/L, respectively [13,14]. Given that our study was based on postoperative complications, we sought to identify new critical values for the NLR and platelet count. To this end, we used the Youden index rather than p-values to determine the critical values for the NLR and platelet count, which are a function of sensitivity and specificity and are widely used to determine the cutoff values of various indicators [24]. Due to the high specificity (78.9%) and low sensitivity (45.3%) of the platelet count, as well as the high specificity (92.9%) and low sensitivity (48.8%) of the NLR, a low preoperative platelet count and a low NLR cannot rule out the risk of developing AL. This suggests that even if patients with esophageal cancer exhibit low preoperative platelet counts or NLR levels, undetected factors may still contribute to the occurrence of postoperative AL. The COP-NLR score exhibits superior predictive value for AL following esophageal cancer surgery compared to platelet count and NLR alone.

Some studies have suggested that patients with a high preoperative or postoperative NLR may be at a significantly increased risk of surgical complications [25,26]. Although the exact reason for the above observation is not yet fully understood, the underlying reason may be related to the physiological state reflected by an elevated NLR, that is, a high neutrophil count and a low lymphocyte count. The activation of neutrophils leads to the generation of reactive oxygen species from reduced nicotinamide adenine dinucleotide phosphate (NADPH), which plays a crucial role in tissue damage. At the same time, the platelet-activating factor released by neutrophils is toxic to endothelial cells and can trigger the release of granzymes, thereby interfering with the normal healing of local wounds. As inflammatory diseases progress, the number of neutrophils gradually increases. Given that neutrophils rely on the glycolytic pathway for energy metabolism, this enables them to function even in low-oxygen environments. It is worth noting that neutrophils consume greater amounts of oxygen than other cell types. This often exacerbates the hypoxia within the inflammatory area and further aggravates postoperative edema and inflammatory responses at the anastomotic site, thus accelerating the development of AL [27]. By contrast, lymphocytes play an important role in alleviating inflammation and promoting the healing process. Normally, the number of lymphocytes decreases as inflammatory diseases progress, probably leading to a blockage of collagen synthesis in the extracellular matrix and thereby weakening the healing ability of the tissue. Chiarelli et al. analyzed data from 306 patients who underwent intestinal resection and anastomosis and demonstrated an independent correlation between low preoperative lymphocyte counts and increased AL incidence and in-hospital mortality [28]. In addition, they found that low postoperative lymphocyte counts were associated with an increased risk of AL [28]. Platelets are differentiated from megakaryocytes and play a role in various stages of inflammation. Preoperative elevated platelet counts can induce a hypercoagulable state in the body that causes reduced tissue blood supply, leading to related complications [29,30]. Moreover, elevated platelets may lead to arterial and venous microembolism in the tubular stomach and proximal esophagus, which affects the anastomotic blood supply and results in the occurrence of AL [31]. Based on these findings, it was hypothesized that systemic inflammation might have an impact on postoperative complications in patients with esophageal cancer. Our study showed that NLR, in combination with platelet count, can be a potent predictor of surgical complications.

As a retrospective study involving multiple surgeons, this research was subject to inherent bias. Furthermore, the lack of systematically recorded surgical technique variables, such as open versus minimally invasive approaches, limited our ability to directly assess their impact on AL. While this study primarily evaluated the short-term predictive utility of the COP-NLR score for AL, it did not comprehensively assess the potential long-term implications of AL on survival outcomes or quality of life in esophageal cancer patients. Despite these limitations, our study demonstrated that the COP-NLR score is capable of functioning as an independent predictor for the occurrence of AL after esophageal cancer resection. Furthermore, the study indicated that preoperative anti-inflammatory treatment could lower the risk of AL in patients with elevated COP-NLR. To further validate the significance of COP-NLR in predicting postoperative complications, larger and more rigorously designed prospective studies need to be conducted in the future.

## Conclusions

This study demonstrated that COP-NLR is an objective predictor of AL after esophageal cancer resection. A comprehensive understanding of various risk factors for AL before surgery, including those reported here and in previous studies, may help prevent high-risk patients from developing AL. Further research is needed to better understand the mechanisms underlying the relationship between high COP-NLR scores and AL occurrence.

## References

[1] Sung H, Ferlay J, Siegel RL, Laversanne M, Soerjomataram I, Jemal A, Bray F (2021). Global Cancer Statistics 2020: GLOBOCAN estimates of incidence and mortality worldwide for 36 cancers in 185 countries. CA Cancer J Clin.

[2] van der Werf LR, Busweiler LA, van Sandick JW, van Berge Henegouwen MI, Wijnhoven BP (2020). Reporting national outcomes after esophagectomy and gastrectomy according to the Esophageal Complications Consensus Group (ECCG). Ann Surg.

[3] Low DE, Kuppusamy MK, Alderson D (2019). Benchmarking complications associated with esophagectomy. Ann Surg.

[4] Kuppusamy MK, Low DE (2020). Evaluation of international contemporary operative outcomes and management trends associated with esophagectomy: a 4-year study of >6000 patients using ECCG definitions and the online Esodata database. Ann Surg.

[5] Mao Y, Gao S, Wang Q (2020). Big data analysis on clinical epidemiological characteristics and surgical treatment profile of esophageal cancer in China (Article in Chinese). Chin J Oncol.

[6] Meng H, Wang Z, Cao H (2017). Progress of influence factors and solutions of esophagogastric anastomotic leak in the perioperative period. Chin J Clin Thorac Cardiovasc Surg.

[7] Duan X, Bai W, Ma Z, Yue J, Shang X, Jiang H, Yu Z (2020). Management and outcomes of anastomotic leakage after McKeown esophagectomy: a retrospective analysis of 749 consecutive patients with esophageal cancer. Surg Oncol.

[8] Kassis ES, Kosinski AS, Ross P Jr, Koppes KE, Donahue JM, Daniel VC (2013). Predictors of anastomotic leak after esophagectomy: an analysis of the society of thoracic surgeons general thoracic database. Ann Thorac Surg.

[9] Messager M, Warlaumont M, Renaud F, Marin H, Branche J, Piessen G, Mariette C (2017). Recent improvements in the management of esophageal anastomotic leak after surgery for cancer. Eur J Surg Oncol.

[10] Celik S, Almalı N, Aras A, Yılmaz Ö, Kızıltan R (2016). Intraoperatively testing the anastomotic integrity of esophagojejunostomy using methylene blue. Scand J Surg.

[11] Blencowe NS, Strong S, McNair AG, Brookes ST, Crosby T, Griffin SM, Blazeby JM (2012). Reporting of short-term clinical outcomes after esophagectomy: a systematic review. Ann Surg.

[12] Feng JF, Huang Y, Chen QX (2014). The combination of platelet count and neutrophil lymphocyte ratio is a predictive factor in patients with esophageal squamous cell carcinoma. Transl Oncol.

[13] Ishizuka M, Nagata H, Takagi K, Iwasaki Y, Kubota K (2013). Combination of platelet count and neutrophil to lymphocyte ratio is a useful predictor of postoperative survival in patients with colorectal cancer. Br J Cancer.

[14] Ishizuka M, Oyama Y, Abe A, Kubota K (2014). Combination of platelet count and neutrophil to lymphocyte ratio is a useful predictor of postoperative survival in patients undergoing surgery for gastric cancer. J Surg Oncol.

[15] Zhang H, Zhang L, Zhu K (2015). Prognostic significance of combination of preoperative platelet count and neutrophil-lymphocyte ratio (COP-NLR) in patients with non-small cell lung cancer: based on a large cohort study. PLoS One.

[16] Nakahira M, Sugasawa M, Matsumura S (2016). Prognostic role of the combination of platelet count and neutrophil-lymphocyte ratio in patients with hypopharyngeal squamous cell carcinoma. Eur Arch Otorhinolaryngol.

[17] Lin YH, Chang KP, Lin YS, Chang TS (2017). Pretreatment combination of platelet counts and neutrophil-lymphocyte ratio predicts survival of nasopharyngeal cancer patients receiving intensity-modulated radiotherapy. Onco Targets Ther.

[18] Tsujino T, Komura K, Ichihashi A (2017). The combination of preoperative platelet count and neutrophil lymphocyte ratio as a prognostic indicator in localized renal cell carcinoma. Oncotarget.

[19] Nakayama M, Gosho M, Hirose Y (2018). Modified combination of platelet count and neutrophil "to" lymphocyte ratio as a prognostic factor in patients with advanced head and neck cancer. Head Neck.

[20] Dolan RD, McSorley ST, Horgan PG, Laird B, McMillan DC (2017). The role of the systemic inflammatory response in predicting outcomes in patients with advanced inoperable cancer: systematic review and meta-analysis. Crit Rev Oncol Hematol.

[21] Radulescu D, Baleanu VD, Padureanu V (2020). Neutrophil/lymphocyte ratio as predictor of anastomotic leak after gastric cancer surgery. Diagnostics (Basel).

[22] Paliogiannis P, Deidda S, Maslyankov S (2020). Blood cell count indexes as predictors of anastomotic leakage in elective colorectal surgery: a multicenter study on 1432 patients. World J Surg Oncol.

[23] Al Lawati Y, Alkaaki A, Luna JL (2021). The predictive value of inflammatory biomarkers in esophageal anastomotic leaks. Ann Thorac Surg.

[24] Youden WJ (1950). Index for rating diagnostic tests. Cancer.

[25] Makal GB, Yıldırım O (2020). Are the C-reactive protein/albumin ratio (CAR), neutrophil-to-lymphocyte ratio (NLR), and platelet-to-lymphocyte ratio (NLR) novel inflammatory biomarkers in the early diagnosis of postoperative complications after laparoscopic sleeve gastrectomy?. Obes Res Clin Pract.

[26] Vulliamy P, McCluney S, Mukherjee S, Ashby L, Amalesh T (2016). Postoperative elevation of the neutrophil: lymphocyte ratio predicts complications following esophageal resection. World J Surg.

[27] Foppa C, Ng SC, Montorsi M, Spinelli A (2020). Anastomotic leak in colorectal cancer patients: new insights and perspectives. Eur J Surg Oncol.

[28] Chiarelli M, Achilli P, Tagliabue F (2019). Perioperative lymphocytopenia predicts mortality and severe complications after intestinal surgery. Ann Transl Med.

[29] Wojtukiewicz MZ, Sierko E, Hempel D, Tucker SC, Honn KV (2017). Platelets and cancer angiogenesis nexus. Cancer Metastasis Rev.

[30] Haemmerle M, Stone RL, Menter DG, Afshar-Kharghan V, Sood AK (2018). The platelet lifeline to cancer: challenges and opportunities. Cancer Cell.

[31] Aiolfi A, Bona D, Bonitta G, Bonavina L (2023). Effect of gastric ischemic conditioning prior to esophagectomy: systematic review and meta-analysis. Updates Surg.

